# Metformin Attenuates Renal Fibrosis in a Mouse Model of Adenine-Induced Renal Injury Through Inhibiting TGF-β1 Signaling Pathways

**DOI:** 10.3389/fcell.2021.603802

**Published:** 2021-02-04

**Authors:** Hao Yi, Chunling Huang, Ying Shi, Qinghua Cao, Jason Chen, Xin-Ming Chen, Carol A. Pollock

**Affiliations:** ^1^Kolling Institute, Sydney Medical School-Northern University of Sydney, Royal North Shore Hospital, St Leonards, NSW, Australia; ^2^Department of Anatomical Pathology, Royal North Shore Hospital, St Leonards, NSW, Australia

**Keywords:** metformin, renal fibrosis, adenine-induced renal injury, transforming growth factor β1 signaling pathways, animal model

## Abstract

It is well-known that all progressive chronic kidney disease (CKD) is pathologically characterized by tubulointerstitial fibrosis process. Multiple studies have shown the critical role of inflammation and fibrosis in the development of CKD. Hence strategies that target inflammatory and fibrotic signaling pathways may provide promising opportunities to protect against renal fibrosis. Metformin has been used as the first-line glucose-lowering agent to treat patients with type 2 diabetes mellitus (T2DM) for over 50 years. Accumulating evidence suggests the potential for additional therapeutic applications of metformin, including mitigation of renal fibrosis. In this study, the anti-fibrotic effects of metformin independent of its glucose-lowering mechanism were examined in an adenine -induced mouse model of CKD. Expressions of inflammatory markers MCP-1, F4/80 and ICAM, fibrotic markers type IV collagen and fibronectin, and the cytokine TGF-β1 were increased in adenine-induced CKD when compared to control groups and significantly attenuated by metformin treatment. Moreover, treatment with metformin inhibited the phosphorylation of Smad3, ERK1/2, and P38 and was associated with activation of the AMP-activated protein kinase (AMPK) in the kidneys of adenine-treated mice. These results indicate that metformin attenuates adenine-induced renal fibrosis through inhibition of TGF-β1 signaling pathways and activation of AMPK, independent of its glucose-lowering action.

## Introduction

Chronic kidney disease (CKD) is a global public health problem. All patients with CKD gradually lose kidney function, with the rate of functional decline varying depending on the disease and patient co-morbidity. When kidney impairment becomes evident there are limited effective treatments available. Current strategies slow the progression of CKD by controlling the underlying cause, including glucose control in Type 1 or type 2 diabetes, treatment of high blood pressure, specific therapies for glomerulonephritis, interstitial nephritis, polycystic kidney disease, relief of obstruction of the urinary tract and treatment of recurrent kidney infection, etc. When CKD progresses to end-stage kidney failure, dialysis and kidney transplantation are required which usually results in significant associated health and social needs, personal loss of independence, a decline in functional capacity and burdens on the health, and societal support systems. Despite tremendous efforts focused on finding efficient therapies that target the progression of tubulointerstitial fibrosis, few therapies are available.

Metformin is the most widely accepted first-line treatment to lower blood glucose levels in patients who have type 2 diabetes mellitus. In addition to its role in lowing blood glucose levels, recent reports suggested it has anti-oncogenic (Leone et al., 2014), cardio-protective (Xiao et al., 2010), and anti-inflammatory effects (Kita et al., 2012). Metformin can attenuate cyclosporine A-induced renal fibrosis in rats (Lin et al., 2019), modulate immune cell infiltration into the kidney during unilateral ureteral obstruction (UUO) in mice (Christensen et al., 2019), significantly reduces renal fibrosis induced by folic acid (Lee et al., 2018; Yi et al., 2018) and ameliorates diabetic nephropathy in a rat model of low-dose streptozotocin-induced diabetes (Zhang et al., 2017), which suggest an antifibrotic effect as well as superior safety and relatively low risk of side effects. However, multiple complex mechanisms as well as different amplifying risk factors are involved in the development of renal fibrosis. Metformin use is currently limited to patients with Type 2 diabetes mellitus and normal renal function or stage 1–3 CKD. Hence there is a need to more fully understand the benefits of metformin in CKD independent of diabetes mellitus and in the presence of significant renal pathology where the magnitude of benefit may be even greater. The adenine induced CKD animal model was developed by Yokozawa et al. (1982). It is well-reported that oral administration of adenine in mice causes classic morphological, biochemical and histopathological alterations in the kidneys, which mimic pathological changes of CKD in humans (Ortiz et al., 2000; Eddy et al., 2012). Previous studies have shown that adenine induces renal functional impairment, myofibroblast activation, sterile inflammatory responses and accumulation of cellular matrix proteins (including collagens and fibronectin) in the renal interstitium. Its mechanism of toxicity has been extensively evaluated with many similarities to human tubulointerstitial pathology (Kashioulis et al., 2018; Abellán et al., 2019; Gong et al., 2019; Ichida et al., 2020; Neven et al., 2020; Sieklucka et al., 2020). Hence this model has been widely used as an animal model of tubulointerstitial kidney disease (Jia et al., 2013; Mishima et al., 2015; Bobeck et al., 2017). This study was undertaken to define the renal protective role of metformin in adenine-induced renal injury. Given the robustness of the development of tubulointerstitial fibrosis, it is an ideal model to assess glucose-independent mechanisms of metformin induced renoprotection and adds to the body of knowledge regarding the benefit of metformin in CKD. Hence, in this study, the renoprotective properties of metformin were explored in a mouse model of adenine-induced renal injury.

## Materials and Methods

### Animal Studies

Eight-week-old male C57BL/6 mice (Kearns Facilities, Kolling Institute), weighing ~20–25 g, were randomly divided into four experimental groups: (1) Control group, (2) Control with Metformin, (3) Adenine, and (4) Adenine with Metformin. Mice were assigned to receive either 4 mg adenine in 200 μl water every day for 21 days, or water alone by oral gavage. Adenine was delivered to mice through oral gavage to avoid the variability of the effect of adenine due to the differential food intake amongst mice. The mice received metformin (0.4 mg/ml) in their drinking water immediately coincident with adenine treatment. The consumption of daily intake of water for each mouse was recorded. No significant differences in water intake were noted between the groups. After treatment with/without metformin for 21 days, a 24-h urine was collected before the animals were sacrificed. The Albuwell M kit and the Creatinine Companion kit (Exocell Inc., Philadelphia, PA) were used to analyze the 24-h urine albumin and creatinine (Philadelphia, PA).

This experiment was conducted according to the recommendations of the National Health and Medical Research Council of Australia and was approved by the Northern Sydney Local Health District Animal Ethics Committee (RESP/17/163).

### Histology and Immunostaining

Paraffin-embedded kidney sections were used for immunohistochemistry staining. After blocking at room temperature for 10 min, the sections were incubated with the diluted primary antibodies (Dako CA) against anti-type IV collagen (1:500), anti-fibronectin (1:500), and anti-TGF-β1(1:500) at 4°C overnight. After incubation with secondary antibodies, sections were developed with DAB (Dako, CA) before being counterstained with hematoxylin. The sections were then quantified using Image J software (Huang et al., 2014). Masson's trichrome staining (American MasterTeck, Lodi, CA) was used to assess tubulointerstitial injury, which was blindly scored using Photoshop software. Interstitial fibrosis, tubular dilation, atrophy, cast formation, or inflammatory cell infiltration were considered as being indicative of interstitial fibrosis (Farris et al., 2011; Martin-Sanchez et al., 2017).

### RNA Isolation and RT-PCR Analysis

Bioline RNA Mini Kit (Bioline, NSW) was used to extract total RNA from mice kidney tissues. The iScript cDNA Synthesis Kit (Bio-Rad) was used to synthesize the cDNA, which was used for quantitative real-time PCR using the SYBR green PCR master mix kit (Invitrogen, CA) with the intron-spanning primers as shown in Table 1. The qPCR was run on ABI-Prism-7900 Sequence Detection System (Applied Biosystems). The quantitation of the mRNAs was performed using the 2^_ΔΔ*Ct*^ method with β-actin as the internal control (Livak and Schmittgen, 2001).

**Table 1:** Nucleotide sequences of the primers used for quantitative real time PCR.

**Target**	**Forward (5^**′**^-3^**′**^)**	**Reverse (5^**′**^-3^**′**^)**
Type IV collagen	TTAAAGGACTCCAGGGACCAC	CCCACTGAGCCTGTCACAC
Fibronectin	CCCTATCTCTGATACCGTTGTCC	TGCCGCAACTACTGTGATTCGG
MCP-1	GCCTGCTGTTCACAGTTGC	CAGGTGAGTGGGGCGTTA
F4/80	CCTGGACGAATCCTGTGAAG	GGTGGGACCACAGAGAGTTG
ICAM1	GTGGCGGGAAAGTTCCTG	CGTCTTGCAGGTCATCTTAGGAG
TGF-β1	TCAGACATTCGGGAAGCAGT	ACGCCAGGAATTGTTGCTAT
β-actin	CAGCTGAGAGGGAAATCGTG	CGTTGCCAATAGTGATGACC

### Western Blotting Analysis

Kidney tissue lysates were separated by SDS-PAGE and transferred to nitrocellulose membranes (Amersham). After incubation with primary antibodies including type IV collagen (1:5,000) (Abcam), fibronectin (1:5,000) (Abcam), α-tubulin (1:10,000) (Sigma-Aldrich), p-p38 (1:500) (Cell Signaling), p38 (1:500) (Cell Signaling), ERK1/2 (1:500) (Cell Signaling), p-ERK1/2 (1:500) (Cell Signaling), p-Smad3 (1:500) (Cell Signaling), and p-AMPK (Cell Signaling) at 4°C overnight, the membranes were incubated with HRP-conjugated secondary antibody (1:5,000) (Amersham, Little Chalfont, United Kingdom) for 1 h. The bands were visualized with ECL and analyzed quantitatively by densitometry using LAS-4000 Imaging System (FUJIFILM, Japan).

### Statistical Analysis

Data were expressed as mean ± SEM and analyzed by one-way ANOVA, followed by Tukey post-test analysis for comparison among multiple groups. *P*-values of *p* < 0.05 were considered statistically significant.

## Results

### Metformin Attenuates Adenine-Induced Renal Injury

**Figure 1:**
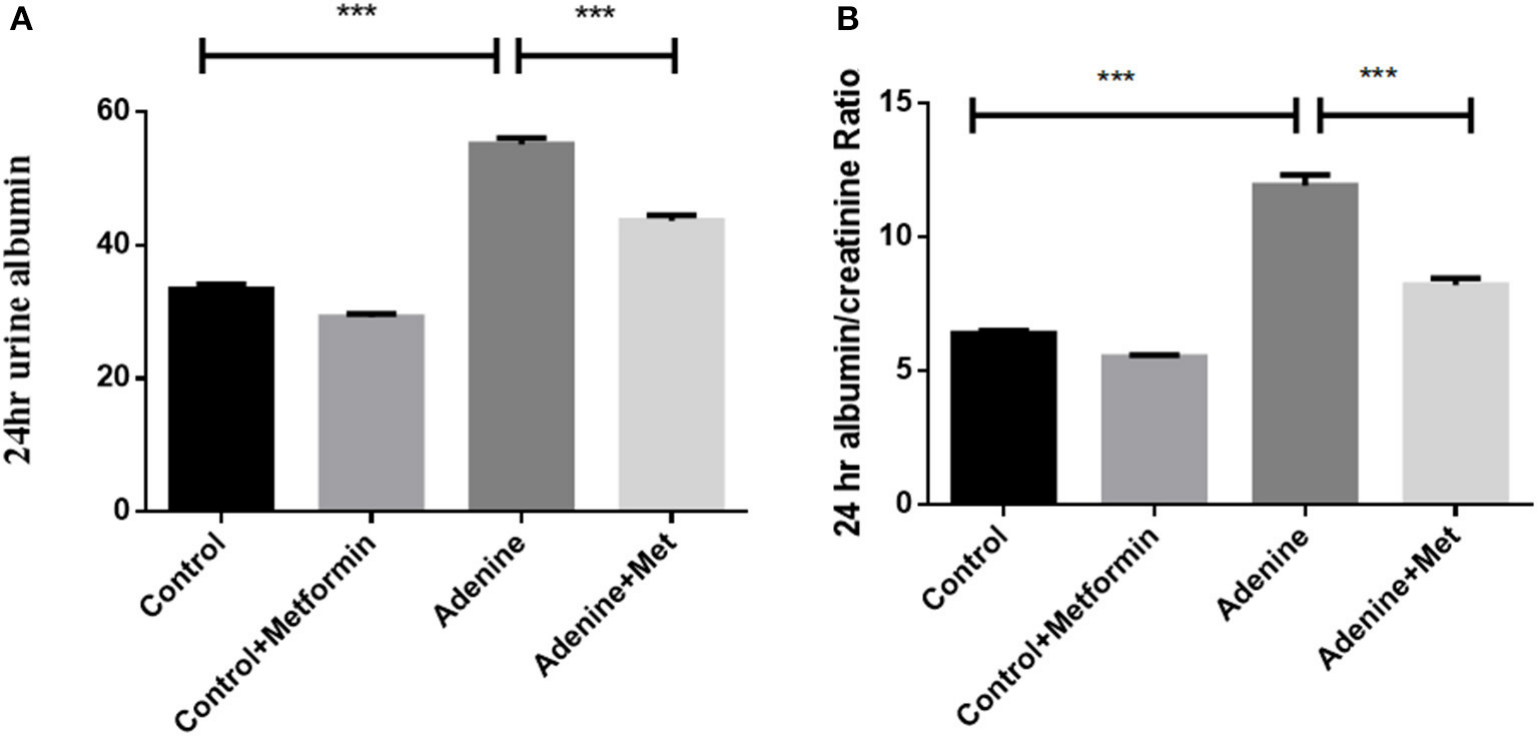
Metformin attenuates adenine-induced renal injury. The 24-h urinary albumin excretion **(A)** and UACR **(B)** were significantly increased in adenine-induced mice compared to the control group, which were attenuated by metformin treatment. Data are expressed as mean ± SEM. ***P < 0.001, *n* = 8.

To determine the effect of metformin on biomarkers of renal pathology, 24-h urine was collected to assess the urinary albumin and albumin to creatinine ratio (UACR). Figure 1A shows an increase in urinary albumin in the adenine exposed group (57.23 ± 0.54 mg/24 h) compared to the control group (33.33 ± 0.93 mg/ 24 h; *P* < 0.001), which was significantly attenuated by metformin treatment (44.43 ± 0.72 mg/24 h) (Figure 1A, *p* < 0.001 vs. adenine alone). Similarly, UACR was significantly increased in adenine exposed group (11.93 ± 0.40 mg/g) compared to control group (6.83 ± 0.05 mg/g) (Figure 1B, *p* < 0.001), which was reduced by metformin treatment (8.20 ± 0.26 mg/g) (Figure 1B, *p* < 0.01 vs. adenine treatment). These data indicated that metformin attenuates adenine-induced renal injury.

### Metformin Reduces Extracellular Matrix Deposition and Tubulointerstitial Fibrosis in a Mouse Model of Adenine-Induced Renal Injury

**Figure 2:**
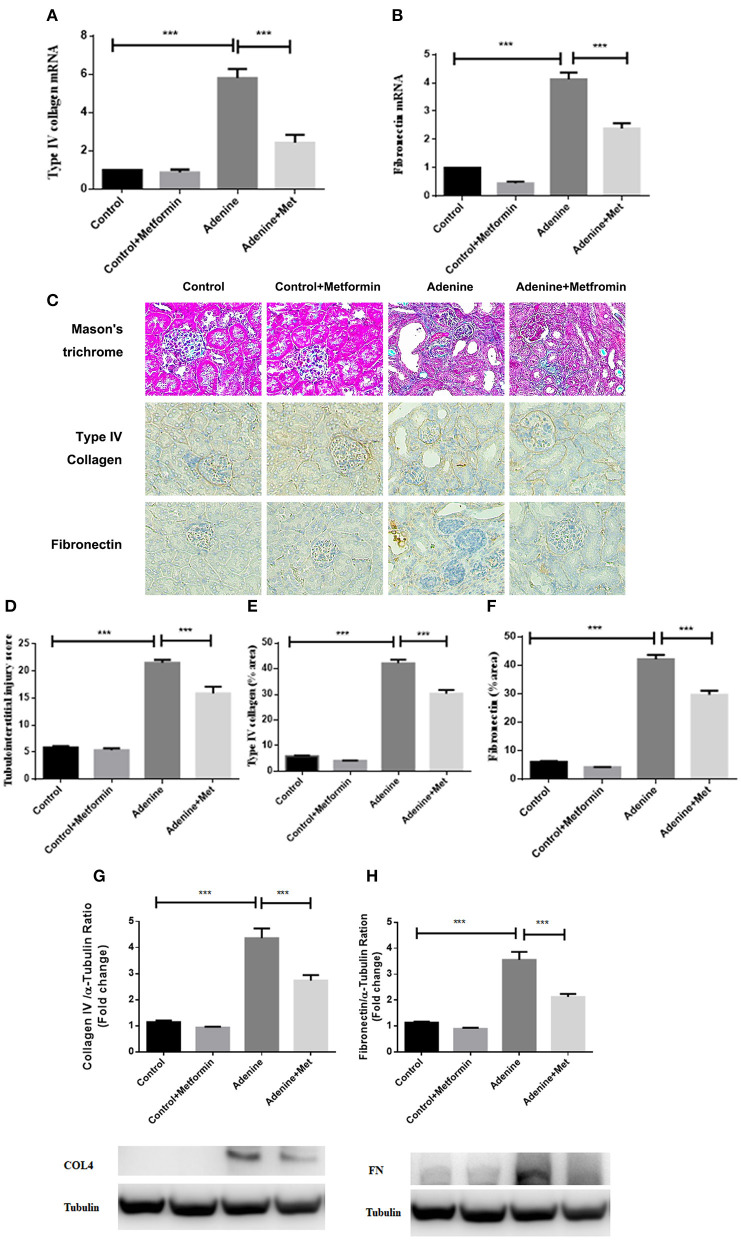
Metformin reduces extracellular matrix deposition and tubulointerstitial fibrosis in a mouse model of adenine-induced renal injury. qRT-PCR results showed that the mRNA level of type IV collagen **(A)** and fibronectin **(B)** were significantly increased in adenine-induced mice compared to the control mice, which were reduced by metformin treatment. Masson's trichrome **(C,D)** and immunohistochemical staining **(E,F)** showed increased tubulointerstitial injury, type IV collagen, and fibronectin expression, which were reduced by metformin treatment. Western blots analysis showed that type IV collagen **(G)** and fibronectin **(H)** expression were significantly increased in adenine-induced mice compared to the control mice, which were reduced by metformin treatment. Data are expressed as mean ± SEM. ***P < 0.001, *n* = 8. Original magnification: ×200.

To define the effect of metformin in fibrotic responses induced by adenine, extracellular matrix type IV collagen and fibronectin mRNA and protein were assessed. RT-PCR analyses showed that the mRNA expression of type IV collagen and fibronectin were significantly increased in kidneys of mice administrated adenine compared to the control group, which was attenuated by metformin treatment (Figures 2A,B, *p* < 0.001). Consistently, immunohistochemistry analyses and western blotting results showed a significantly increased staining of type IV collagen and fibronectin (Figures 2E–H, *p* < 0.001) in kidneys of mice administrated adenine compared to the control group. Metformin treatment reduced type IV collagen and fibronectin deposition in kidneys of adenine exposed mice compared with control mice (Figures 2E–H, *p* < 0.001). Interstitial extracellular matrix deposition was examined by Masson's trichrome staining. A significant increase in tubulointerstitial injury was observed in kidneys of mice administrated adenine compared to the control group (*p* < 0.001), which was attenuated by metformin treatment (Figures 2C,D, *p* < 0.001). These results indicate that metformin reduces extracellular matrix overproduction and renal fibrosis in a model of adenine-induced renal injury.

### Metformin Prevents Inflammatory Responses in a Mouse Model of Adenine-Induced Renal Injury

**Figure 3:**
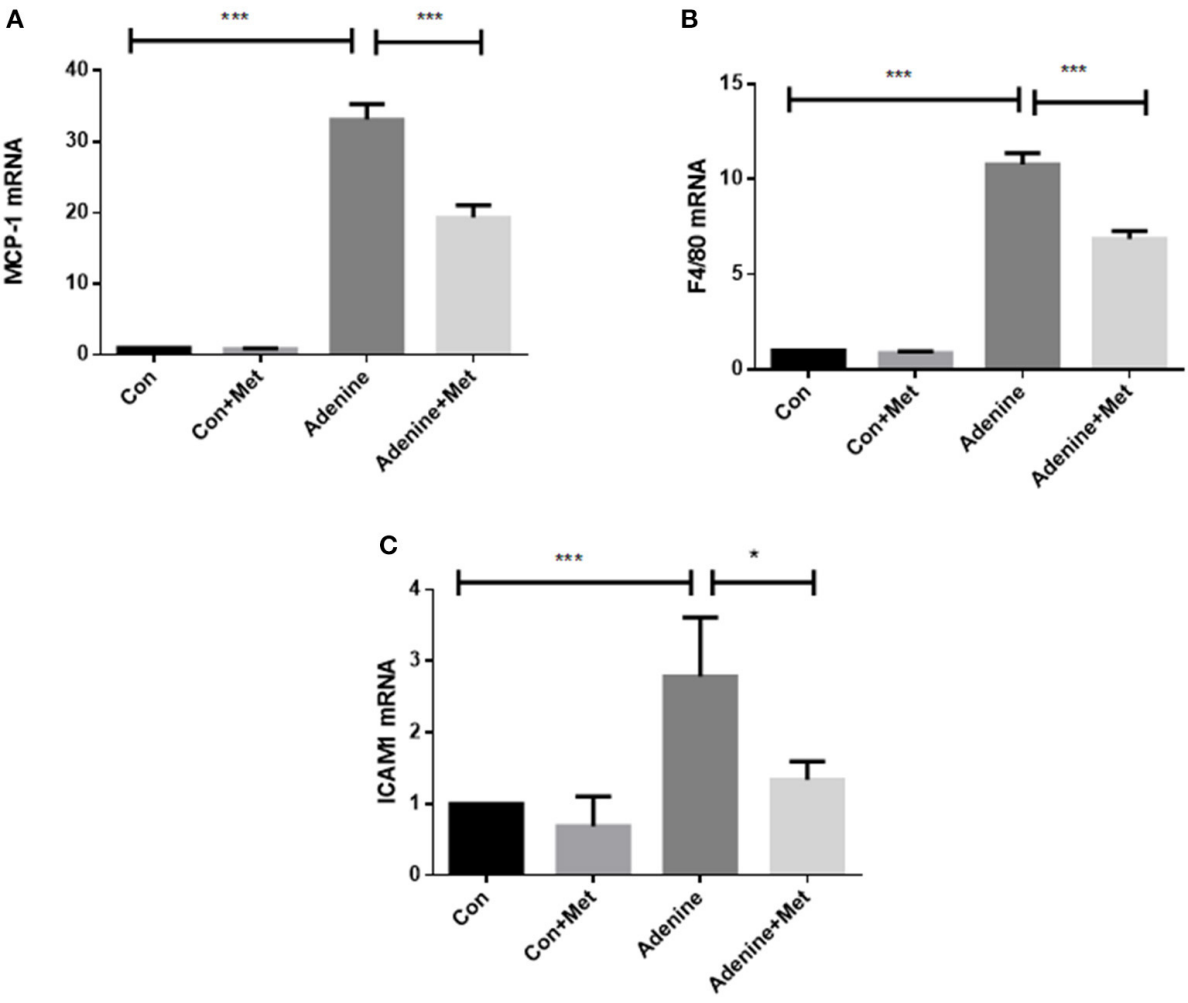
Metformin prevents inflammatory responses in a mouse model of adenine-induced renal injury. qRT-PCR results showed that the mRNA expression of MCP-1 **(A)**, F4/80 **(B)**, and ICAM1 **(C)** were significantly increased in adenine treated mice compared to the control mice, which were reduced by metformin treatment. Data are expressed as mean ± SEM. *P < 0.05, ***P < 0.001, *n* = 8.

It is well-known that chronic inflammation promotes the development of tissue fibrosis. The expression of inflammatory markers including macrophage chemotactic protein (MCP-1) and macrophage activation markers F4/80 and intercellular adhesion molecule 1 (ICAM1) was used to determine the role of metformin in the regulation of inflammation. MCP-1 is considered a critical marker of renal inflammation in models of kidney injury (Doi et al., 2006). The qRT-PCR result showed that MCP-1 level was increased by 32.12-fold in the kidneys of mice administrated with adenine compared to control (Figure 3A, *p* < 0.001). Metformin treatment attenuated adenine induced MCP-1 level in kidneys (Figure 3A, *p* < 0.001). The expression of F4/80 and ICAM1 was increased in the adenine exposed mice compared to the control mice (Figures 3B,C, *P* < 0.001), which was attenuated by metformin treatment (*P* < 0.05). These results suggest that metformin prevents inflammation in adenine induced kidney injury by inhibiting proinflammatory cytokine production and macrophage infiltration.

### Metformin Suppresses Upregulation of TGF-β1 in a Mouse Model of Adenine-Induced Renal Injury

**Figure 4:**
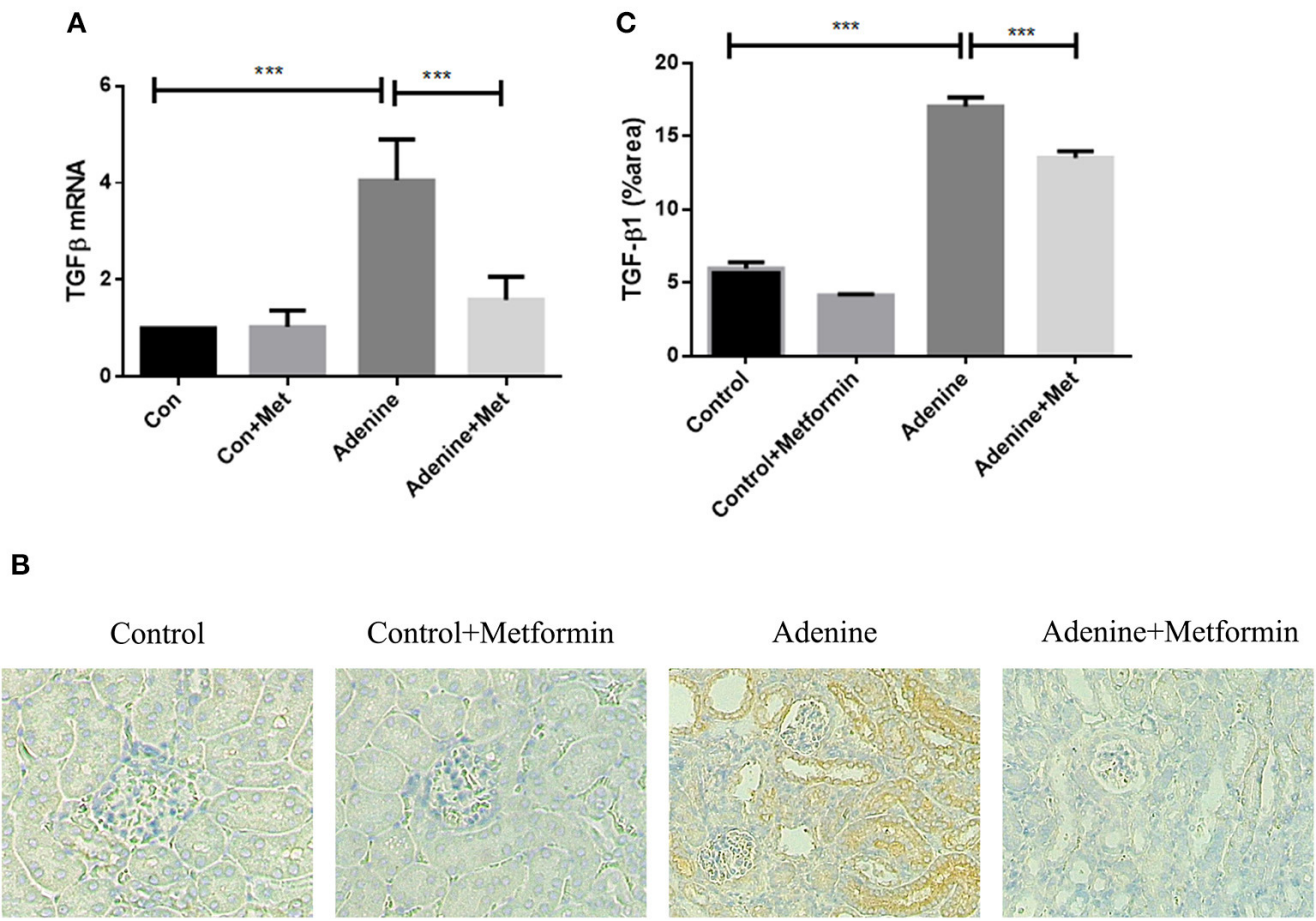
Metformin suppresses the upregulation of TGF-β1 expression in the mouse model of adenine-induced renal injury. qRT-PCR **(A)** and immunohistochemical staining **(B,C)** results demonstrated that the expression of TGF-β1 was significantly increased in adenine-induced mice compared to the control mice, which was suppressed by metformin treatment. Data are expressed as mean ± SEM. ***P < 0.001, *n* = 8. Original magnification: ×200.

TGF-β1, a key profibrotic growth factor, is crucial in the development of most forms of kidney disease. Hence TGF-β1 expression was examined in the kidneys of mice exposed to adenine +/– metformin. mRNA expression of TGF-β1 was significantly upregulated in kidneys of mice exposed to adenine compared with the control group (Figure 4A, *p* < 0.001), which was attenuated in metformin-treated mice (Figure 4A, *p* < 0.001). Consistently, immunohistochemical analyses also demonstrated that TGF-β1 protein expression was significantly increased in kidneys of mice exposed to adenine compared to the control group (Figures 4B,C, *p* < 0.001), which was limited by metformin treatment (Figures 4B,C, *p* < 0.001). The results demonstrate that metformin inhibits overexpression of TGF-β1 mRNA and protein in the kidneys of mice with adenine induced kidney injury.

### Metformin Suppresses TGF-β1 Signaling Pathways Through Inhibiting Activation of Smad3, ERK1/2 and p38 in a Mouse Model of Adenine-Induced Renal Injury

**Figure 5:**
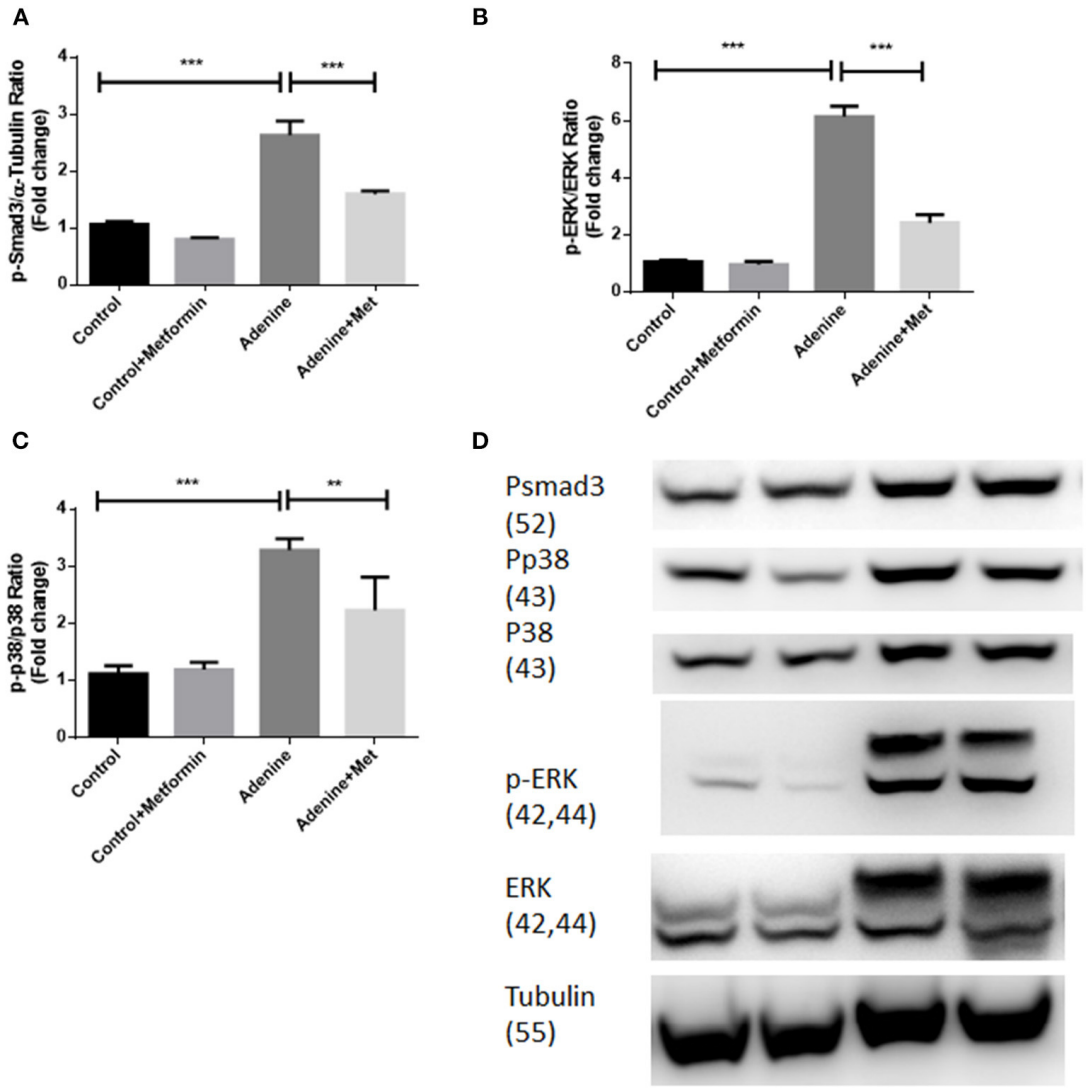
Metformin suppresses TGF-β1 signaling pathways through inhibiting activation of Smad3, ERK1/2, and p38 in a mouse model of adenine-induced renal injury. Western blot results demonstrated that metformin suppressed adenine induced phosphorylation of p-Smad3 **(A,D)**, p-ERK1/2 **(B,D)**, and p-P38 expression **(C,D)** in adenine treated mice. Data are expressed as mean ± SEM. **P < 0.01, ***P < 0.001, *n* = 8.

To examine if metformin inhibits TGF-β1 downstream signaling pathways in kidneys of mice with adenine induced kidney injury the phosphorylation of Smad3, ERK1/2, and p38 were examined in kidney tissues using western blot analyses. Figure 5 showed that the phosphorylation of Smad3, ERK1/2, and p38 (Figures 5A–C, *p* < 0.001) were all significantly increased in kidneys of mice exposed to adenine compared to the control group, which was inhibited by metformin treatment (Figures 5A,B, *p* < 0.001, Figure 5C, *p* < 0.01). These data demonstrate that metformin confers renoprotection in the model of adenine-induced renal injury by inhibiting TGF-β1 signaling pathways.

### Metformin Activated AMPK in a Mouse Model of Adenine-Induced Renal Injury

**Figure 6:**
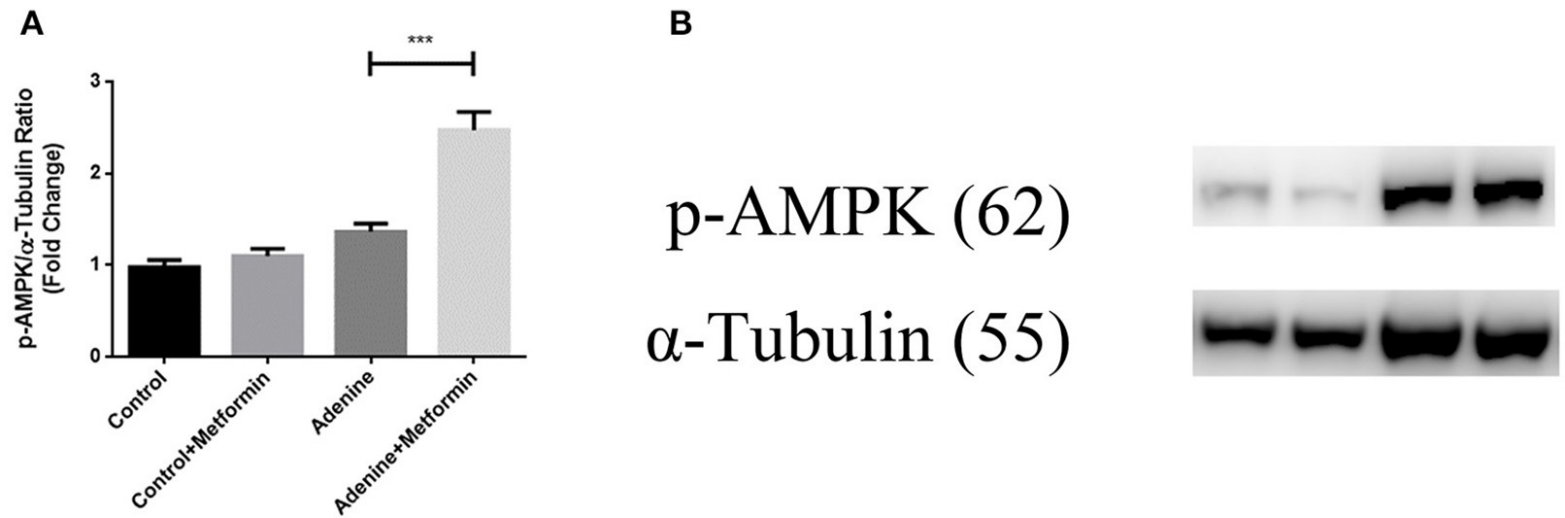
Metformin activated AMPK in a mouse model of adenine-induced renal injury. The result of western blot results showed that metformin activated AMPK **(A,B)** in metformin-treated mice. Results are presented as mean ± SEM. ***P < 0.001, *n* = 8.

It is well-known that metformin acts through both AMPK-dependent and AMPK-independent mechanisms. To examine if the AMPK-dependent mechanisms were activated in mice with adenine induced kidney injury, the phosphorylation of AMPK was examined in kidney tissues using western blot analyses. Figure 6 showed that although adenine did not reduce the phosphorylation of AMPK compared to control, metformin significantly increased p-AMPK compared to control and the adenine exposed groups (Figure 6A, *p* < 0.001). These data demonstrated that metformin may protect against renal injury through AMPK-dependent mechanisms.

## Discussion

This study was undertaken to determine if metformin attenuates renal injury in a non-diabetic model of tubulointerstitil renal injury and to elucidate the possible mechanisms. The present study uniquely demonstrates that metformin ameliorated extracellular matrix deposition and inflammation in an adenine-induced CKD mouse model. Furthermore, the study showed that metformin exerted its antifibrotic effect through suppression of TGF-β1 expression and downstream TGF-β1 signaling pathways.

Metformin, an adenosine monophosphate-activated protein kinase (AMPK) activator, is a commonly used drug to control blood glucose levels in patients with type 2 diabetes mellitus. Metformin has also been reported to limit liver, cardiac, lung and renal fibrosis (Xiao et al., 2010; Schuppan and Kim, 2013; Sato et al., 2016; Yi et al., 2018). Metformin's direct renoprotective role, independent of its glucose lowing effect, has been demonstrated in a high-fat diet, low-dose streptozotocin-induced rat model of diabetic kidney disease (Zhang et al., 2017). Metformin markedly attenuated characteristic renal pathological lesions and reduced glomerular basement membrane thickness, which was accompanied by decreased TGF-β1 expression (Zhang et al., 2017). However, the role of metformin on non-diabetic kidney disease models of renal injury is less well-studied and to date only in limited non-diabetic models of kidney disease. A prior study demonstrated that metformin suppressed macrophage infiltration, expression of markers of inflammation, extracellular matrix proteins, TGF-β1 expression, and interstitial fibroblast activation in obstructed kidneys which led to the conclusion that metformin prevents renal inflammation and fibrosis in mice (Cavaglieri et al., 2015). It has also been reported that metformin attenuated renal fibrosis in UUO mice due to inhibition of Ang-II-induced extracellular matrix production in renal fibroblasts through the inhibition of ERK signaling (Shen et al., 2016). A further study has demonstrated that the protective effects of metformin are mediated by AMPKα2-dependent and AMPKα2-independent targeting of TGF-β1 downstream signaling (Feng et al., 2017). Recently, metformin has been shown to modulate immune cell infiltration into the kidney in UUO mice, which limits subsequent fibrotic responses (Christensen et al., 2019). Metformin has also been shown to inhibit lipid accumulation and fibrosis in the kidneys of mice with nephropathy, and to increase fatty acid oxidation via modulation of Acetyl-CoA carboxylase by AMPK (Lee et al., 2018). Our previous study has also demonstrated that metformin treatment attenuated TGF-β1 induced inflammatory and fibrotic responses in human proximal tubular cells (HK2 cells) and folic acid-induced renal injury in C57BL mice (Guan et al., 2018; Malsin and Kamp, 2018; Wu et al., 2018; Yi et al., 2018; Yoshida et al., 2019).

Collectively, the anti-fibrotic role of metformin in diabetic kidney disease, obstructive nephropathy, and folic acid-induced nephropathy has been well-documented. Renal fibrosis is the common pathological endpoint of end-stage chronic kidney disease. The mechanisms of chronic kidney disease are complex due to different upstream causes and impacted upon by co-morbidities in an individual. The glucose independent role of metformin in the development of adenine-induced nephropathy in mice was assessed and confirmed in our study. Adenine significantly upregulated expression of type IV collagen, and fibronectin, and overall extracellular matrix in kidneys of mice with adenine-induced renal injury, which were significantly reversed by metformin treatment. These results add to the literature suggesting that metformin exerts anti-fibrotic effects in chronic kidney disease (Zhang et al., 2017; Lee et al., 2018; Yi et al., 2018; Christensen et al., 2019).

Sterile inflammation has an important role in initiating renal fibrosis (Lv et al., 2018). MCP-1 is the most studied mediator of renal inflammation (Tesch, 2008). MCP-1 promotes proliferation, infiltration, and production of more cytokines and chemokines of inflammation cells. F4/80 is widely used as a marker of macrophage infiltration. The increased expression of F4/80 in kidneys indicates active inflammatory responses and has been well-accepted as being inherent in renal injury (Cao et al., 2015; Wang et al., 2017). Intracellular adhension molecule-1 (ICAM1), which is also known as cluster of differentiation 54 (CD54), is a protein with a signal-transducing function considered to increase proinflammatory pathways. Activation of ICAM1 recruits inflammatory immune cells such as macrophages to maintain a pro-inflammatory environment for leukocyte infiltration. Thus, ICAM1 is an important marker for macrophage infiltration in kidney tissue. In this study, metformin inhibited adenine-induced overexpression of MCP-1, F4/80, and ICAM1 in kidneys, which confirmed the anti-inflammatory role of metformin in adenine-induced renal injury.

It is well-documented that TGF-β1 signaling pathways play a central role in renal interstitial fibrosis. The upregulation of TGF-β1 signaling pathways has been considered to be fundamental to all causes of kidney disease in both experimental models and human kidney disease (Bottinger and Bitzer, 2002; Wang et al., 2005). The TGF-β1 signaling pathways include Smad and non-Smad pathways, which are involved in *many* cellular processes. Smad signaling is the major pathway of TGF-β1 signaling in renal fibrosis, and Smad3 has been shown to mediate renal fibrosis in various mouse models of chronic kidney diseases (Meng et al., 2015; Chen et al., 2018). TGF-β1 non-Smad pathways signaling molecules include the extracellular signal-regulated kinases (ERKs), c-Jun amino-terminal kinase (JNK), p38 the mitogen-activated protein kinase (MAPK), the IκB kinase (IKK), phosphatidylinositol-3 kinase (PI3K) and Akt, as well as Rho family GTPases. The non-Smad signaling molecules contribute to the physiological responses as stand-alone pathways or together with Smads (Zhang, 2009, 2017). It is well-documented that in addition to regulating transcription through the phosphorylation of Smad2 and Smad3 and formation of a Smad2/3/4 complex, TGF-β1 also mediates other non-Smad signaling pathways (Meng et al., 2016). TGF-β1 activates ERK, p38 MAPK, and JNK to mediate renal fibrosis (Meng et al., 2016), which is independent of the Smad pathway. In our study, the results showed that metformin inhibited TGF- β1 expression as well as Smad and non-Smad signaling pathways as demonstrated by inhibition of phosphorylation of Smad3, ERK1/2, and p38 in kidneys of mice with adenine-induced renal injury. These data indicate that the antifibrotic effects of metformin are at least mediated through TGF-β1 and its downstream Smad and non-Smad signaling pathways.

It is well-known that AMPK is a pivotal molecule that prevents or delays the process of fibrogenesis and AMPK exerts comprehensive protective effects against fibrosis in various organs and tissues (Jiang et al., 2017). The AMPK-dependent and AMPK-independent mechanisms of metformin have been well-studied (Kalender et al., 2010; Vincent et al., 2015). The data in this study have shown that metformin inhibits the TGFb pathway through suppressing both smad and non-smad pathway, which indicated the effects were AMPK-independent. Furthermore, a recent study has also shown that metformin could inhibit TGF-β-induced collagen production in mice by activating AMPK (Lu et al., 2015). Collectively these data are consistent with the demonstrated in this study.

In conclusion, the present study suggests that metformin can improve renal function and protect against chronic renal injury induced by adenine through inhibiting TGF-β1 signaling pathways and potentially by increasing the phosphorylation of AMPK. These results, together with other studies (Zhang et al., 2017; Lee et al., 2018; Yi et al., 2018; Christensen et al., 2019), confirm metformin exerts renoprotection independent of its glucose lowing effect in non-diabetic kidney disease. These results suggest an antifibrotic role for metformin in diverse forms of chronic kidney disease, thus warranting therapeutic evaluation in clinical settings.

## Data Availability Statement

The raw data supporting the conclusions of this article will be made available by the authors, without undue reservation.

## Ethics Statement

The animal study was reviewed and approved by Animal Ethics Committee Northern Sydney Local Health District.

## Author Contributions

HY, CP, and X-MC did conception and design of experiments. HY, CH, YS, and QC performed experiments and analyzed data. JC helped to analyses the IHC images. HY drafted the manuscript. All authors contributed to manuscript revision, read and approved the final version of manuscript.

## Conflict of Interest

The authors declare that the research was conducted in the absence of any commercial or financial relationships that could be construed as a potential conflict of interest.
